# The Role of Adiponectin in the Pathogenesis of Metabolic Disturbances in Patients With Schizophrenia

**DOI:** 10.3389/fpsyt.2020.605124

**Published:** 2021-01-20

**Authors:** Cynthia Yi-An Chen, Kah Kheng Goh, Chun-Hsin Chen, Mong-Liang Lu

**Affiliations:** ^1^Department of Psychiatry, Wan-Fang Hospital, Taipei Medical University, Taipei, Taiwan; ^2^Psychiatric Research Center, Wan-Fang Hospital, Taipei Medical University, Taipei, Taiwan; ^3^Department of Psychiatry, School of Medicine, College of Medicine, Taipei Medical University, Taipei, Taiwan

**Keywords:** adiponectin, schizophrenia, antipsychotics, metabolic disturbance, leptin, ghrelin, time-dependent drug effect

## Abstract

Antipsychotic-induced metabolic disturbance is a common adverse event occurring in patients treated with antipsychotic drugs. The mechanisms underlying metabolic dysregulation are complex, involving various neurochemical and hormonal systems, the interaction of genetic and lifestyle risk factors, and the antipsychotic drug prescribed. Recently, there has been increasing interest in the relationship between antipsychotic-induced metabolic disturbances and body weight regulatory hormones such as adiponectin. Adiponectin, an adipocyte-derived protein related to insulin sensitivity, weight gain, and anti-inflammation, has attracted great attention because of its potential role of being a biomarker to predict cardiovascular and metabolic diseases. Previous studies regarding the effects of antipsychotics on blood adiponectin levels have shown controversial results. Several factors might contribute to those inconsistent results, including different antipsychotic drugs, duration of antipsychotic exposure, age, sex, and ethnicity. Here we summarize the existing evidence on the link between blood adiponectin levels and metabolic disturbances related to antipsychotic drugs in patients with schizophrenia. We further discuss the effects of individual antipsychotics, patients' gender, ethnicity, age, and treatment duration on those relationships. We propose that olanzapine and clozapine might have a time-dependent biphasic effect on blood adiponectin levels in patients with schizophrenia.

## Introduction

Schizophrenia is a major mental disease causing substantial impairment and burden (1). Patients with schizophrenia have higher rates of physical comorbidity and mortality compared to the general population (2, 3). In patients with schizophrenia, the risks of cardiovascular disease and metabolic disease are 2- and 5-fold higher, respectively, than in the general population (4, 5). People with schizophrenia have a 2–3 times increase in standard mortality ratio for all-cause mortality (6, 7). Metabolic and cardiovascular diseases play an important role in these tragedies (8). High mortality and morbidity in patients with schizophrenia may also be partially attributed to unhealthy behaviors, such as smoking, substance abuse, lack of exercise, and poor dietary habits (9). Data from drug-naïve populations suggested that people with schizophrenia may be susceptible to metabolic disturbances (10). However, these findings remain controversial (11, 12) and further investigations are needed to distinguish whether these findings are contributed to genetic factors or unhealthy lifestyle behaviors. Furthermore, antipsychotic drugs, especially some second-generation antipsychotics (SGAs) and low-potency first-generation antipsychotics (FGAs), also increase the risk of metabolic abnormalities (8).

**Figure 1:**
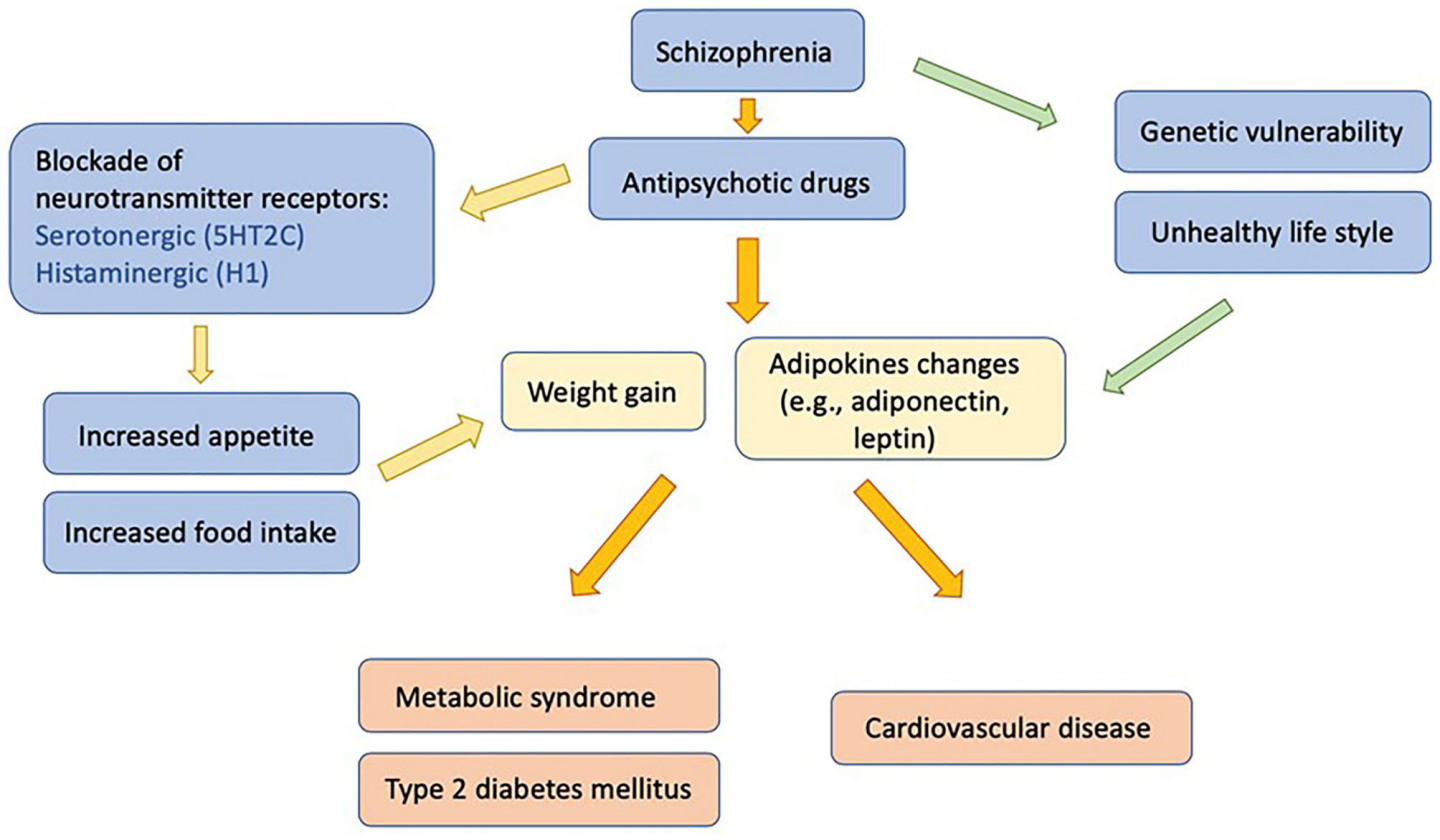
Influences of antipsychotic medication on adipokines, metabolic disturbances, and associated morbidities in patients with schizophrenia.

The underlying mechanisms of antipsychotic-induced metabolic abnormalities are complex, involving various neurochemical and hormonal systems, the interaction of genetic and lifestyle risk factors, as well as the antipsychotic drug prescribed. The blockades of serotonin 5-hydroxytryptamine 2C (5-HT_2C_) and histamine 1 (H_1_) receptors by antipsychotics have been associated with weight gain and metabolic dysregulations. Several studies found that adipokines, biologically active cytokines secreted by adipose tissue, may also play an important role (13). Among those adipokines, adiponectin plays a crucial role in causing the comorbid conditions of schizophrenia and metabolic dysregulation (Figure 1) (14). Previous reports have suggested differing results of the effects of antipsychotics on adiponectin levels. Several factors might contribute to these differing results, including different antipsychotic drugs, as well as patients' duration of antipsychotic exposure, age, sex, and ethnicity. In this review, we intend to summarize the role of adiponectin in patients comorbid with schizophrenia and metabolic disturbance. We also further discuss the effects of individual antipsychotics, gender, ethnicity, age, and treatment duration on adiponectin levels under those comorbid conditions.

## Antipsychotic Drugs and Side Effects

Antipsychotic drugs are the mainstay pharmacological treatment for patients with schizophrenia (15). Antipsychotic drugs are divided into FGAs and SGAs. FGAs work through dopamine D_2_ receptor blockade and are effective in treating positive symptoms of schizophrenia, such as hallucinations and delusion. Extrapyramidal symptoms (EPS) are common neurologic side effects of antipsychotic medications. The risk of EPS varies remarkably among individual antipsychotics. The efficacy of SGAs in improving positive and negative symptoms, cognition, as well as daily functioning has been proven (16–19). The antagonist action in both 5-HT_2_ and D_2_ receptors are important for the therapeutic efficacy of SGAs. A recent network meta-analysis revealed that most antipsychotics have similar therapeutic efficacy but different side effect profiles (20). With few exceptions, only clozapine, amisulpride, zotepine, olanzapine, and risperidone are more effective in reducing overall symptoms of schizophrenia as compared with other antipsychotics (20). There are significant differences between antipsychotics concerning metabolic dysregulations, with olanzapine and clozapine exhibiting the worst profiles (21).

Metabolic adversities of antipsychotics have become a new challenge for clinicians (22). Several interconnected mechanisms of metabolic disturbance have been suggested, including increased appetite/food intake, physical inactivity and unhealthy lifestyle, as well as patients' characteristics such as gender and genetic variants (23). The binding of SGAs to 5-HT_2C_ and H_1_ receptors has also been associated with weight gain and metabolic alterations (24). Metabolic adversities can predict cardiovascular events, which are the main reason for higher mortality rates in patients with schizophrenia as compared to those in the general population (6, 25). Among commonly used antipsychotics, olanzapine, and clozapine are usually related to more metabolic side effects while aripiprazole, brexpiprazole, cariprazine, lurasidone, and ziprasidone establish better metabolic profiles (21). Lurasidone, cariprazine, aripiprazole, and brexpiprazole even show improved metabolic parameters in terms of glucose level, low-density lipoprotein (LDL) cholesterol level, and high-density lipoprotein (HDL) cholesterol level (21). Therefore, identifying metabolic abnormalities and early prevention of possible cardiovascular events in patients with schizophrenia become an important task. Several potential targets are proposed to be biomarkers of metabolic disturbances in patients with schizophrenia (26–32). Among these, blood adiponectin level is a promising biomarker of cardiovascular and metabolic diseases, especially in antipsychotic-medicated patients (28, 33, 34).

## The Adiponectin Signaling System

Adiponectin is secreted diurnally by adipocytes into the circulatory system, accounting for about 0.01–0.05% of total serum proteins (35), and functions as a messenger connecting adipose tissue and other organs (36). Human adiponectin comprises 244 amino acids and can be separated into three multimers: low molecular weight (LMW) form, trimer; middle molecular weight (MMW) form, hexamer; and high molecular weight (HMW) form, 12–18 monomers (37). After entering the circulating system, these multimers seldom interchange between different forms and present distinct biochemical characteristics (38). Previous studies support that adiponectin receptors are widely expressed in the brain (39, 40). Adiponectin can cross the blood-brain barrier and act directly on adiponectin receptors in the cortex, hypothalamus, and pituitary gland (39).

Adiponectin is an adipose-derived protein related to insulin sensitivity, weight gain, and anti-inflammation, attracting great attention recently because of its potential role of being a biomarker to predict metabolic syndrome (41, 42). Other than insulin-sensitizing and anti-inflammatory properties, adiponectin also shows anti-apoptotic, proangiogenic, anti-atherogenic, proadipogenic (37, 43, 44), and even anti-depressive effects (45, 46). In an animal study, treatment with adiponectin reduces blood levels of free fatty acid, triglycerides, and glucose (47). Higher blood adiponectin levels are associated with improvements in executive function and global cognition (48, 49), as well as lower risks of myocardial infarction, coronary artery disease, and other cardiovascular events (50). Failure to up-regulate adiponectin production may play an important role in developing insulin resistance, diabetes mellitus, metabolic syndrome, and atherosclerosis (51).

Leptin, insulin-like growth factor, and growth hormone are positively related to adiponectin gene expression, while tumor necrosis factor-α (TNF-α) and interleukin-6 (IL-6) act contrarily (37). However, the influence on adiponectin gene expression by insulin is differing, with some studies observing increased gene expression caused by insulin (52, 53) and some reporting down-regulation (54).

Antipsychotic drugs acting as antagonists at dopamine D_2_ receptors can stimulate prolactin release (55). Accumulating evidence suggests that prolactin may be linked to the development of type 2 diabetes mellitus through insulin signaling pathway (56). Furthermore, prolactin may act as an adipokine to suppress the production of adiponectin by adipocytes and influence energy homeostasis (57).

Two adiponectin receptors – adiponectin receptor 1 (AdipoR1) and adiponectin receptor 2 (AdipoR2) – prevalently express in the liver, muscle, heart, adipose tissue, pancreas, and the brain (58). Among multiple signaling molecules activated by adiponectin, adenosine monophosphate-activated protein kinase (AMPK) plays a major role in the downstream signaling (39). Adiponectin also works on AdipoR1, inducing extracellular calcium influx necessary for activating calcium/calmodulin-dependent protein kinase kinase (CaMKK) (58). Through binding to AdipoR2, adiponectin activates the expression of peroxisome proliferator-activated receptor alpha (PPARα) ligands, as well as increases glucose and lipid metabolism (51). Multiple studies have proven insulin-sensitizing effect of adiponectin, which mostly takes place in the liver and skeletal muscle (59–61). Adiponectin reduces triglyceride in the liver and muscle, but induces beta-oxidation in skeletal muscle via AMPK activation, leading to improvement in insulin sensitivity (37). Beside AMPK involvement, multiple signaling mechanisms through p38-mitogen-activated protein kinases (p38-MAPK), PPARα, and adaptor protein, phosphotyrosine interacting with PH domain and leucine zipper 1 (APPL1) are reported (39). Several studies proposed that impaired adiponectin action is a hallmark of obesity-linked diseases, through the mechanism of hypoadiponectinemia and down-regulation of adiponectin receptors (58).

Previous evidence suggested that peripheral effects of adiponectin are mainly mediated by HMW adiponectin (39). But increasing studies proposed that LMW adiponectin might be the active forms in the central nervous system, as LMW adiponectin has been detected in human cerebrospinal fluid (62, 63). Further studies are necessary to explore the role of adiponectin in the brain and the effects of different adiponectin multimers.

### High Molecular Weight Adiponectin

Although blood LMW adiponectin, MMW adiponectin, and HMW adiponectin levels are all lower in patients with metabolic syndrome, HMW adiponectin is a stronger candidate to predict insulin resistance and metabolic syndrome among different multimers of adiponectin (64). Studies have shown decreased blood HMW adiponectin level resulting in the progression of metabolic syndrome and even type 2 diabetes mellitus (65–67). Blood HMW adiponectin level is proposed to be a better predictor of the progression to metabolic syndrome compared to total blood adiponectin level or HMW/total adiponectin ratio (67, 68). HMW adiponectin has the strongest relationship with insulin sensitivity as compared with LMW adiponectin or HMW/total adiponectin ratio (68). Chen et al. also reported that only HMW adiponectin level is correlated with insulin sensitivity rather than total adiponectin level in patients with schizophrenia (26).

A study by Lee et al. has reported lower HMW adiponectin levels in patients with schizophrenia compared to non-psychiatric controls (33). Both schizophrenia and non-psychiatric groups have shown that lower HMW adiponectin levels are associated with higher body mass index, worse risk for coronary heart disease, higher number of metabolic syndrome criteria, greater insulin resistance, lower levels of HDL cholesterol, and higher levels of high sensitivity C-reactive protein (CRP) (33).

### Leptin and Leptin/Adiponectin Ratio

Leptin is also an adipose-derived protein related to appetite, obesity, energy balance, insulin resistance, and other metabolic parameters. Elevated blood leptin level is associated with insulin resistance and the risk of metabolic syndrome. Hyperleptinemia can also be a risk factor for cardiovascular disease (64, 69). Elevated blood leptin levels are observed in patients with schizophrenia, particularly in those taking SGAs (70). Leptin might be one of the factors involving in the mechanisms of SGAs related to body weight gain and adiposity (71). Although both adiponectin and leptin levels have the potential to be biomarkers for predicting metabolic syndrome (41, 72–74), recent studies have suggested leptin/adiponectin ratio (L/A ratio) as a stronger candidate as compared with adiponectin and leptin alone (27, 73). The L/A ratio is positively correlated with most of the metabolic parameters, including body weight, body mass index, waist circumference, cholesterol level, triglycerides level, LDL level, insulin level (27), and most prominently, insulin resistance (27, 75, 76). In addition, the L/A ratio is positively correlated to the blood levels of CRP and amyloid A (SAA), which are obesity-related inflammatory markers (77). This finding implies that the L/A ratio can be a biomarker to predict the severity of adipose tissue dysfunction and cardiometabolic risk (78).

Regarding as a biomarker of metabolic abnormality, the cut-off point of the L/A ratio is still inconclusive. Frühbeck et al. have proposed to define the L/A ratio value below 1 as normal, between 1 and 2 as moderate, above 2 as severe cardiometabolic risk in the general population (78). Another study by Larsen et al. suggested that the cut-off value at 1.88 can detect increased risks of early obesity-related metabolic disturbances (79). A study by Chen et al. reported that the L/A ratio can better discriminate against schizophrenia patients with and without metabolic syndrome than using the value of either leptin or adiponectin (27). They have proposed 0.61 as the optimal cut-off value of the L/A ratio for metabolic syndrome in patients with schizophrenia (27).

### Ghrelin

Ghrelin, a 28-amino-acid orexigenic peptide, is predominantly produced by the stomach (80). In humans, ghrelin stimulates growth hormone release, modifies energy homeostasis, increases appetite, and leads to weight gain (81). In addition, an increase in blood ghrelin levels dysregulates adipose-liver interaction, increases the free fatty acid released from adipose tissue, as well as alters adiponectin and cytokine secretion (82). The action of ghrelin contributes to the development of the metabolic syndrome and type 2 diabetes mellitus (83). Zhang et al. proposed that SGAs have a time-dependent effect on serum ghrelin levels, with an initial increase by the acute effect of SGAs in the first week, followed by a down-regulation due to negative feedback from SGA-induced weight gain in 2–6 weeks, and finally a return to baseline or above during long-term SGA treatment (84). But the effect of antipsychotics on ghrelin level varies depending on individual antipsychotics, patients' age, gender, dietary pattern, lifestyle, physical and psychiatric co-morbidity, co-medication, as well as other confounding factors (84, 85). In addition, acylated ghrelin and desacylated ghrelin are the two main forms of serum ghrelin, demonstrating different roles in energy homeostasis (83). A study by Wu et al. revealed that acylated/desacylated ghrelin ratio is a better biomarker for metabolic syndrome than other ghrelin parameters in olanzapine-treated patients with schizophrenia (30).

## Effects of Antipsychotics on Adiponectin

### Drug-Naïve Patients With Schizophrenia

Metabolic adversities are well-known adverse effects of antipsychotic drugs. It is still debatable whether metabolic syndrome in patients with schizophrenia is induced by antipsychotics or schizophrenia *per se*. Available evidence suggests that the clinical efficacy and side effects of antipsychotics vary from patient to patient. The large individual variability of metabolic abnormality of antipsychotics can be attributed to multifactorial mechanisms, in which genetic factors may be essential. A twin study has highlighted the possibility of genetic factors for weight gain after antipsychotic treatment (86). Several genes have been reported to be strongly associated with metabolic syndrome in patients with schizophrenia, such as the fat mass and obesity-associated gene (FTO), leptin and leptin receptor genes (LEP, LEPR), methylenetetrahydrofolate reductase (MTHFR) gene, catechol-o-methyl transferase (COMT) gene, insulin-induced gene (INSIG) 2, sterol regulatory element-binding transcription factor 2 (SREBF2) gene, dopamine receptor D2 gene (DRD2), and the serotonin receptor 2C gene (HTR2C) (87–90).

Some studies showed that even drug-naïve patients with schizophrenia are more vulnerable to metabolic adversities than healthy controls, with the manifestation of glucose intolerance and insulin resistance before antipsychotic use (91–94). Increased visceral fat deposition has also been discovered in drug-naïve schizophrenia patients (95). On the other hand, a meta-analysis by Bartoli et al. revealed that blood adiponectin levels in drug-naïve patients with schizophrenia are not different from those of healthy controls (96). These findings suggest that metabolic disturbances of drug-naïve schizophrenia patients are caused by a mechanism other than the adiponectin pathway.

### Individual Antipsychotics

Antipsychotic drugs are widely used in several mental illness entities and give a different extent of risk for antipsychotic-induced metabolic dysregulation. Almost all antipsychotic drugs, including FGAs and SGAs, cause metabolic side effects with quantitative and qualitative differences (97). Recently published data have ranked clozapine and olanzapine as having the highest risk of metabolic adversity, followed by paliperidone, quetiapine, risperidone, and haloperidol in the middle, and aripiprazole, lurasidone, and ziprasidone with the lowest risk (14, 98). A recent meta-analysis reported that schizophrenia *per se* is not associated with lower blood adiponectin levels (96). Schizophrenia patients with metabolic syndrome have lower blood adiponectin levels as compared to those without metabolic syndrome, and blood adiponectin levels are decreased as the number of metabolic syndrome components is increased (99, 100). Individuals with schizophrenia taking SGAs have lower blood adiponectin levels than normal controls (96). Furthermore, differential effects exist on blood adiponectin levels among various antipsychotic drugs.

#### Haloperidol

Haloperidol has the least potential to cause weight gain among antipsychotic drugs (21). Pre-clinical and clinical studies proposed that haloperidol seemed not to affect blood adiponectin level. *In vitro* study found that haloperidol does not cause adiponectin (ADIPOQ) gene expression (101). *In vivo* study revealed that 12-week haloperidol medication in Sprague Dawley rats shows lower weight gain and similar blood adiponectin levels compared to controls (102). A study by Perez-Iglesias et al. found that body weights increase and blood adiponectin levels remain unchanged in drug-naive psychotic patients after a 1-year haloperidol treatment (103). Raposo et al. also reported that body weights and blood adiponectin levels are similar in patients with schizophrenia after a 9-month haloperidol medication (104).

#### Risperidone

Risperidone shows a moderate risk of metabolic dysregulation among antipsychotics (21). *In vitro* study reported that risperidone increases the expression of ADIPOQ gene (105). An *in vivo* study revealed that risperidone increases adiponectin mRNA in male Sprague-Dawley rats (106). A study by Perez-Iglesias et al. showed that blood adiponectin levels increase after a one-year risperidone treatment in drug-naive psychotic patients (103). Wampers et al., and Sugai et al. also found that blood adiponectin levels increase in schizophrenia patients receiving risperidone treatment for 3 and 12 months, respectively (107, 108).

#### Olanzapine

Olanzapine exhibits the highest risk of metabolic dysregulation among antipsychotics (21). An *in vitro* study reported that 7-day olanzapine exposure does not affect total adiponectin expression or multimer composition of secreted protein (109). Another *in vitro* study found that an 11-day olanzapine exposure has moderate effects on ADIPOQ gene expression (101). In an animal study using female rats, olanzapine-induced hyperphagia has been found to cause weight gain, increased adiposity, and subsequent insulin resistance, although the latter may be alleviated by the compensatory response to produce adiponectin (110). Astudy by Cooper et al. revealed that male olanzapine-medicated rats have neither hyperphagia, reduced weight gain, enhanced visceral adiposity, nor increased blood adiponectin level (111), whereas another study has shown an increased body weight and subcutaneous fat deposition in male rats receiving higher doses of olanzapine (112). This trend of sex difference has been found in many animal studies receiving different antipsychotics (113–115).

In human studies, a single dose of 10 mg olanzapine does not cause any effect on blood adiponectin levels in healthy controls (116). Increased blood levels of triglyceride and adiponectin have been found after an 8-day olanzapine use in non-psychotic healthy male subjects (117). After receiving a 4-week treatment of olanzapine, patients with schizophrenia have remarkable weight gains but no changes in blood adiponectin levels (118). Schizophrenia patients under olanzapine for more than 3 months have decreased blood adiponectin levels (70, 107). Base on those findings we suggest that olanzapine might have a time-dependent effect on blood adiponectin levels in patients with schizophrenia.

#### Clozapine

An *in vitro* study revealed that clozapine can induce the expression of ADIPOQ gene (101, 105). An animal study by von Wilmsdorff et al. has shown weight gain and increased blood adiponectin levels in male rats receiving clozapine, as compared with controls (102).

In a human study, a 6-week administration of clozapine has shown no changes in blood adiponectin levels among patients with childhood-onset schizophrenia (119). Patients with schizophrenia receiving long-term clozapine medication have lower blood adiponectin levels than healthy subjects (70). In patients with schizophrenia receiving clozapine for at least 3 months, blood adiponectin levels are negatively associated with weight gain and metabolic parameters after receiving clozapine treatment (28). Being independent of age and body mass index, hypoadiponectinemia is a potential biomarker of the metabolic syndrome in schizophrenia patients receiving clozapine treatment (28). Similar to olanzapine, clozapine has a time-dependent effect on blood adiponectin levels in patients with schizophrenia.

#### Aripiprazole

The results of the effect of aripiprazole on adiponectin are inconsistent. An *in-vitro* study reported that aripiprazole increases blood adiponectin levels in the supernatants on mouse fibroblast cultures (120). Another *in-vitro* study revealed that aripiprazole does not change the mRNA expression of ADIPOQ gene on human subcutaneous adipose tissue (121). The study by Gao et al. reported that patients with first episode schizophrenia receiving a 24-week aripiprazole treatment have lower blood adiponectin levels than those before treatment (122). The study by Wang et al. reported that after aripiprazole augmentation for 8 weeks, patients treated with olanzapine have decreased body weight and blood triglyceride levels, but increased adiponectin blood levels (123). Further studies are warranted to investigate the effect of aripiprazole on adiponectin production.

## Gender, Ethnic, and Age Effects on Adiponectin Levels

In the majority of clinical studies, female patients receiving antipsychotics tend to gain more weight and be diagnosed with metabolic syndrome when compared to male counterparts (124). The vulnerability of female rodents to weight gain and metabolic side effects of SGAs over male ones is comparable to that found in the clinical studies (110, 111). Gender difference in blood adiponectin levels has also been found. In both animal and human studies, females show higher total and HMW adiponectin levels than males (27, 125–127), which may be contributed to the effect of androgens (126). Since androgens are likely to play an inhibitory role in blood adiponectin level, women are then expected to have higher blood adiponectin levels than men. This trend has also been found in patients with schizophrenia, with male patients presenting lower blood adiponectin level and lower L/A ratio comparing to female patients (27), although not all studies replicated the same results (96). Moreover, Chen et al. revealed that L/A ratio has a stronger predictive ability for metabolic syndrome in male patients than in female counterparts (27). Matsuda et al. reported an association of obesity and decreased plasma adiponectin level only observed in male patients with schizophrenia but not in female patients (128). The correlation of obesity and elevated insulin resistance is also more prominent in the male group than the female one (128).

Ethnic and intra-ethnic variations exist, with higher blood adiponectin levels in Europeans and Aboriginal people as compared with Chinese and South Asians (129). Both South Asians and Aboriginal people show increased insulin resistance when blood adiponectin levels are decreased (129). Another study reported that the impact of body mass index on insulin resistance, CRP, and adiponectin appears greater in Chinese as compared with other major Asian ethnic groups (130). The study by Lee et al. reported that lower blood levels of HMW adiponectin are associated with minority race/ethnicity in patients with schizophrenia (33).

Remarkably, the effect of adiponectin varies in different age groups. Studies in people without schizophrenia have reported higher blood adiponectin levels with aging, proposing that higher blood adiponectin levels in older adults do not reflect better health as they do in younger persons (131). In a healthy middle-aged population (aged 40–59 years), low blood adiponectin levels are associated with a higher risk of coronary heart disease (50). But in people aged 65 and older, higher blood adiponectin levels are found to be associated with an increased risk of coronary heart disease (132, 133). Cohort studies revealed that blood adiponectin levels increase over time in older people and are associated with greater physical disability and mortality (131).

## Time-Dependent Effect of Antipsychotics on Adiponectin Levels

**Figure 2:**
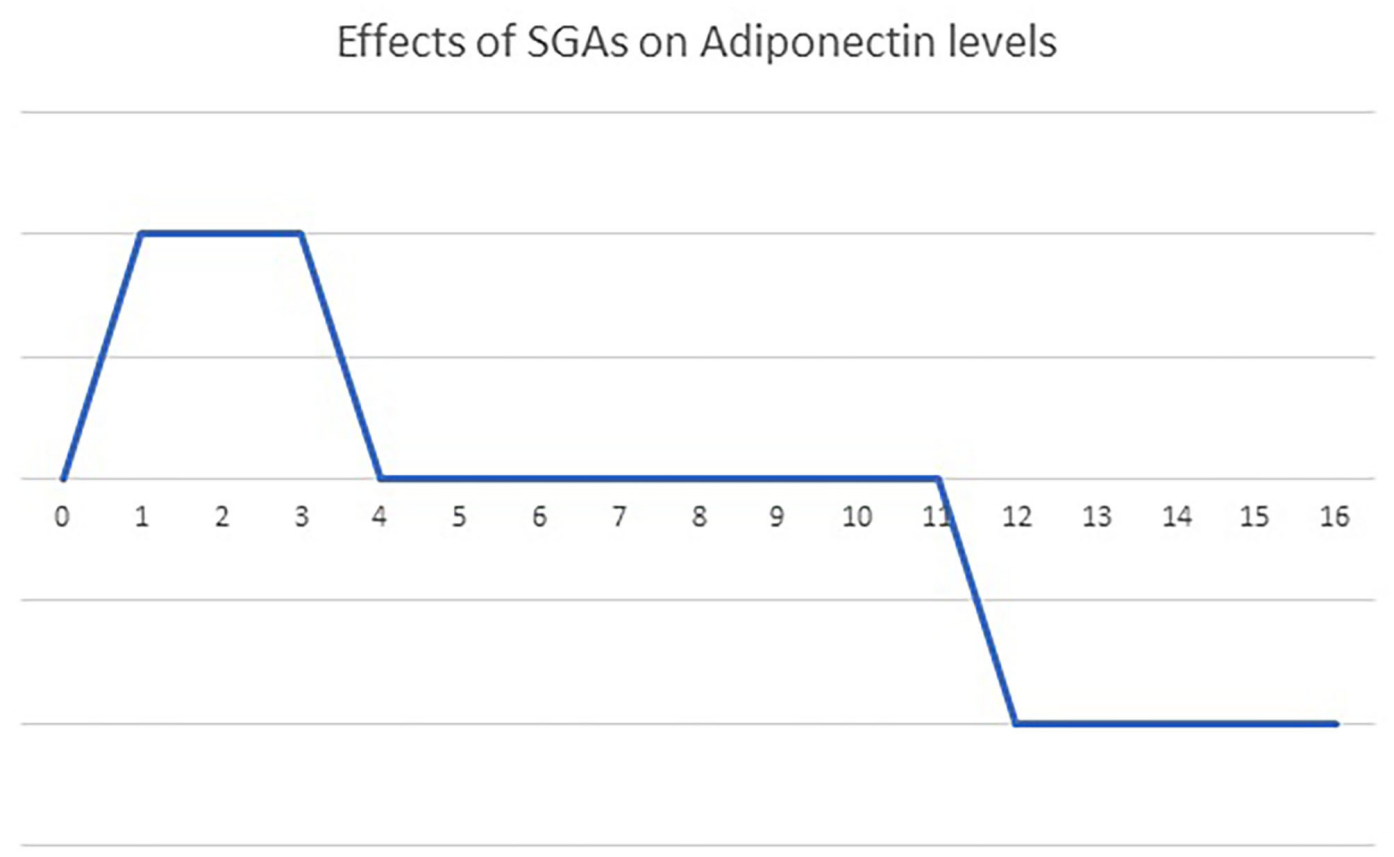
The bi-phasic effects of olanzapine and clozapine on blood adiponectin levels over time. A single dose of olanzapine or clozapine had no influence on blood adiponectin levels. Treatment with olanzapine or clozapine up-regulates blood adiponectin levels acutely (*t* = 1–4 weeks), to compensate for the side effect on glucose homeostasis. Then, a new energy balance equilibrium is re-established during short-term treatment (4–12 weeks), returning blood adiponectin levels to the baseline. Finally, the failure of adiponectin up-regulation pushes blood adiponectin levels further below the baseline after long-term treatment (more than 3 months).

In addition to the individual characteristics of antipsychotic drugs, the duration of antipsychotic medication might also contribute to the inconsistent results of the effect of antipsychotics on blood adiponectin levels (Figure 2). A single dose of 10 mg olanzapine has no effect on blood adiponectin levels in healthy controls (116). After an 8-day olanzapine treatment in healthy males, blood triglyceride levels, insulin resistance, and adiponectin levels increased (117). This up-regulation of adiponectin might compensate for the detrimental effect of olanzapine on insulin sensitivity. After a 4-week olanzapine medication, patients with schizophrenia show weight gains but no changes in blood adiponectin levels (118). Schizophrenia patients receiving olanzapine for more than 3 months have decreased blood adiponectin levels (70, 107). Clozapine also has similar time-dependent effects on blood adiponectin levels. After receiving 6-week clozapine medication, patients with childhood-onset schizophrenia show no changes in blood adiponectin levels (119). After receiving clozapine treatment for more than 3 months, patients with schizophrenia have increased body weight and decreased blood adiponectin levels (28, 70). Srisawasdi et al. also proposed that risperidone treatment decreases blood adiponectin levels in a duration-dependent manner in children and adolescents with autistic spectrum disorders (134).

Apparently, the effect of olanzapine and clozapine on blood adiponectin levels might vary according to the duration of treatment (Figure 2). We propose that olanzapine and clozapine might have a time-dependent biphasic effect on adiponectin levels in patients with schizophrenia. Initially, the up-regulation of adiponectin might compensate for the deleterious effect of olanzapine and clozapine on glucose homeostasis. Then a new energy balance equilibrium is re-established during a short-term treatment, resulting in the return of blood adiponectin levels to the baseline. Finally, the failure of adiponectin up-regulation pushes blood adiponectin levels further below the baseline after long-term treatment.

## Conclusion

Metabolic disturbance is a common side effect of antipsychotic treatment. The mechanisms underlying metabolic dysregulation are complex, involving various neurochemical and hormonal systems, the interaction of genetic and lifestyle risk factors, as well as the antipsychotic drug prescribed. Clinical and preclinical data indicated that failure to upregulate adiponectin production is related to antipsychotic-induced metabolic disturbances. But differential effects on blood adiponectin levels exist among various antipsychotic drugs. Additionally, treatment duration, sex, age, and ethnicity also influence blood levels of adiponectin and the risk of metabolic syndrome. We suggest that olanzapine and clozapine might have a time-dependent biphasic effect on adiponectin levels in patients with schizophrenia. To elucidate the role of adiponectin in antipsychotic-induced metabolic dysregulations, future studies are warranted to investigate the impacts of those factors on energy homeostasis.

In conclusion, recognition of regulatory effects of adiponectin under antipsychotic medication could lead to novel treatment approaches for metabolic disturbances of patients with schizophrenia. As the new era of personalized medications has arrived, potential markers that provide information on possible metabolic risk can help clinicians adjust therapeutic strategy to prevent suffering owing to metabolic dysfunction or even associated premature death. Furthermore, up-regulation of the adiponectin receptor pathway might be a potential therapeutic target for antipsychotic-induced metabolic disturbances.

## Author Contributions

M-LL provided the ideation and writing of the manuscript. CY-AC contributed significantly to the writing of the manuscript. KKG reviewed the literature papers that were used for writing the manuscript. C-HC has made a critical review of the manuscript. All authors contributed to the article and approved the submitted version.

## Conflict of Interest

The authors declare that the research was conducted in the absence of any commercial or financial relationships that could be construed as a potential conflict of interest.
